# Disrupted-in-Schizophrenia 1 (DISC1) Overexpression and Juvenile Immune Activation Cause Sex-Specific Schizophrenia-Related Psychopathology in Rats

**DOI:** 10.3389/fpsyt.2019.00222

**Published:** 2019-04-09

**Authors:** Taygun C. Uzuneser, Jil Speidel, Georgios Kogias, An-Li Wang, Maria A. de Souza Silva, Joseph P. Huston, Iulia Zoicas, Stephan von Hörsten, Johannes Kornhuber, Carsten Korth, Christian P. Müller

**Affiliations:** ^1^Department of Psychiatry and Psychotherapy, University Hospital, Friedrich-Alexander-University Erlangen-Nuremberg, Erlangen, Germany; ^2^Center for Behavioral Neuroscience, Institute of Experimental Psychology, University of Düsseldorf, Düsseldorf, Germany; ^3^Department of Experimental Therapy, Preclinical Experimental Center, Friedrich-Alexander-University Erlangen-Nuremberg, Erlangen, Germany; ^4^Department of Neuropathology, Medical Faculty, Heinrich-Heine-University Düsseldorf, Düsseldorf, Germany

**Keywords:** disrupted-in-schizophrenia 1, schizophrenia, immune activation, synaptic pruning, gene environment interaction, animal model

## Abstract

Synaptic pruning is a critical refinement step during neurodevelopment, and schizophrenia has been associated with overpruning of cortical dendritic spines. Both human studies and animal models implicate disrupted-in-schizophrenia 1 (DISC1) gene as a strong susceptibility factor for schizophrenia. Accumulating evidence supports the involvement of DISC1 protein in the modulation of synaptic elimination during critical periods of neurodevelopment and of dopamine D2-receptor-mediated signaling during adulthood. In many species, synaptic pruning occurs during juvenile and adolescent periods and is mediated by microglia, which can be over-activated by an immune challenge, giving rise to overpruning. Therefore, we sought to investigate possible interactions between a transgenic DISC1 model (tgDISC1) and juvenile immune activation (JIA) by the bacterial cell wall endotoxin lipopolysaccharide on the induction of schizophrenia-related behavioral and neurochemical disruptions in adult female and male rats. We examined possible behavioral aberrations along three major symptom dimensions of schizophrenia including psychosis, social and emotional disruptions, and cognitive impairments. We detected significant gene–environment interactions in the amphetamine-induced locomotion in female animals and in the amphetamine-induced anxiety in male animals. Surprisingly, gene–environment interactions improved social memory in both male and female animals. JIA alone disrupted spatial memory and recognition memory, but only in male animals. DISC1 overexpression alone induced an improvement in sensorimotor gating, but only in female animals. Our neurochemical analyses detected sex- and manipulation-dependent changes in the postmortem monoamine content of animals. Taken together, we here report sex-specific effects of environment and genotype as well as their interaction on behavioral phenotypes and neurochemical profiles relevant for schizophrenia.

## Introduction

Schizophrenia is a severe psychiatric disorder whose symptoms are generally classified into three major groups: positive (psychotic) symptoms, negative symptoms, and cognitive impairments. It is widely accepted that the pathogenesis is frequently driven by the synergistic contribution of several genetic and environmental factors (1). Accumulating evidence implicates the disrupted-in-schizophrenia 1 protein (DISC1) as a strong candidate susceptibility factor for schizophrenia (2–5). Studies about the biological function of DISC1 using yeast two-hybrid screening revealed DISC1 to be an intracellular scaffold protein, interacting with various proteins to regulate neurodevelopmental and synaptic processes (3–5). Synaptic pruning is a key refinement process during neurodevelopment in which the redundant synapses are eliminated and the remaining synapses are strengthened (6). DISC1 has been shown to be critically involved in synaptic pruning, and its malfunction could cause abnormal spine morphology (5). Overpruning of cortical synapses during critical neurodevelopmental periods has been proposed to underlie schizophrenia, as many studies investigating postmortem brain samples of schizophrenic patients have reported reduction of spine density on cortical and hippocampal pyramidal neurons (7–9).

Schizophrenia, as well as many other psychiatric disorders, arise from genetic confounders, but are also strongly influenced by the environmental factors. Such factors include prenatal and postnatal immune activation (10), several forms of stress (11), adolescent cannabis use (12), and late paternal age (13). Monozygotic twin studies also support the involvement of non-genetic contributors. Among these non-genetic contributors, immune activation during critical periods of neurodevelopment is a possible modulator of spine morphology. Microglia cells have been shown to be involved not only in active immune defense but also in spine maintenance and synaptic remodeling in the healthy brain during neurodevelopment (14, 15). Microglia have been shown to make frequent contact with synapses and engulf the dendritic spines *via* phagocytosis during the juvenile period in mice (16). Paolicelli et al. also showed that a transient decrease in microglia surveillance, extension of processes to sample the extracellular space, caused reduced synaptic pruning in mice, further implementing the role of microglia for synaptic maintenance (15). Therefore, in many species, juvenile and early adolescent periods are extremely vulnerable for an immune challenge, which would influence the synaptic elimination process, causing irreversible consequences.

The requirement for the inclusion of female animals in preclinical experiments has been stressed for a long time (17, 18). Investigating both sexes in psychiatric research is being highly promoted (18, 19), as there exist many sex differences in psychiatric disorders relating to prevalence, disease onset, symptoms, comorbidities, and treatment outcome. Schizophrenia similarly shows such gender differences. Compared to female patients, the prevalence of schizophrenia is slightly higher, the onset of symptoms is earlier, cognitive symptoms are more severe, and vulnerability to develop a comorbid substance abuse is significantly higher in males (20, 21).

Various animal models based on DISC1 dysfunction have been generated since its identification as a robust risk factor in schizophrenia (22, 23). However, the effects of DISC1 dysfunction on rodent behavior have been primarily investigated in male animals. Furthermore, among the environmental risk factors of schizophrenia, the immune activation studies focus primarily on the prenatal and neonatal periods, neglecting the critical juvenile period, during which spine morphology is significantly altered. In order to address these issues, we combined a DISC1-transgenic animal model with juvenile immune activation (JIA) in male and female rats to induce a behavioral phenotype relevant to schizophrenia, as well as to investigate possible effects of sex on the phenotype. For this purpose, we used non-mutant full-length human DISC1 overexpression with coding polymorphisms (S704C, L607F) in rats under the control of the Syrian hamster prion protein (PrP) promoter (24, 25). The coding variants S704C and L607F were chosen because they occur frequently in the general population and were shown to be a risk factor for mental disorders (2, 26–28). This animal model was created to mimic DISC1 aggregates observed in a subset of patients with mental illness (29) and thus to represent as an animal model for sporadic forms of DISC1 protein-linked behavioral disorders (25, 30). We induced an immune response by the administration of lipopolysaccharide (LPS), a bacterial cell wall endotoxin, which has been shown to activate the peripheral and central immune system and to induce sickness behavior (31). LPS was administered subchronically between postnatal days 22 and 30, as the synaptic refinement processes have been shown to be actively ongoing during this period (15, 32). Under inflammatory situations induced by LPS, activated microglia have been shown to become more phagocytic, which can cause overpruning during critical time periods (33). We hypothesized that JIA by LPS treatment would over-activate the synaptic pruning processes, and its combination with tgDISC1 could synergistically induce schizophrenia-related behavior in adulthood in female and male rats. Since schizophrenia is implicated in aberrant monoamine neurotransmission especially in the cortical and striatal regions (34) and altered postmortem monoamine profiles have also been observed in tgDISC1 animal models (25, 35), we also investigated the postmortem levels of monoamines in the brain regions that have been commonly associated with schizophrenia.

## Materials and Methods

### Animals

Transgenic male and female Sprague–Dawley rats and their non-transgenic littermates were used. Transgenic animals (tgDISC1) were generated as detailed by Trossbach et al. (25). They contained the full-length, non-mutant human DISC1 as transgene with the polymorphisms S704C and L607F under control of the Syrian hamster prion protein (PrP) promoter. Integration of the transgene was confirmed by quantitative real-time PCR (qRT-PCR). Transgenic test animals contained an allele of inserted DISC1 gene on both homologous chromosomes (homozygous).

Newborn animals were weaned on postnatal day 21, separated by their sexes, and housed as four animals per cage in a temperature-controlled (22 ± 2°C) and humidity-controlled (55 ± 10%) room under a light–dark cycle (light on from 07:00 to 19:00). Food and water were provided *ad libitum*. All experiments were conducted in conformity with the Animal Protection Law of the Federal Republic of Germany as well as the European Communities Council 2010 Directive (2010/63/EU), and were approved by local authorities (Regierung von Mittelfranken).

### Transgene Detection by qRT-PCR

Tail biopsies were digested in a lysis buffer containing 100 mM Tris (pH 8), 5 mM EDTA, 0.2% SDS, 200 mM NaCl, and 100 mg/ml proteinase K. Each tail sample was incubated with 500 µL of lysis buffer overnight with constant shaking (800 rpm) at 50°C. Genomic DNA was precipitated by 100% isopropanol and 70% ethanol followed by centrifugation (30 min, 4°C, 14,000 rpm) and solubilized in pure water.

For the transgene detection, a qRT-PCR was conducted using primers binding the PrP promoter region (forward: 5′-CTGATCTCCAGAAGCCCAAA-3′; reverse: 5′-CAGGCCTATTCCTTGACAGC-3′). For normalization, primers binding the housekeeping gene rat beta-actin were used (forward: 5′-GCAACGCGCAGCCACTGTCG-3′; reverse: 5′-CCACGCTCCACCCCTCTAC-3′). The reaction mixes included genomic DNA samples, SYBR Green I SuperMix (Roche, Mannheim, Germany), FactorQ, distilled water, and either PrP or beta-actin primers. PCR cycle parameters: 10 min at 95°C and then 40 cycles of 10 s at 95°C, 20 s at 60°C, and 30 s at 72°C. The data were analyzed using the LightCycler 480 software (Roche, Mannheim, Germany). DISC1 expression was normalized to the expression of reference gene beta-actin (25).

### Drugs and Treatment Procedure

LPS [strain: *Escherichia coli* (*E. coli*) O111:B4, Sigma Aldrich] was dissolved in saline (SAL). Animals received five i.p. injections of LPS on alternate days between postnatal days 22 and 30, which is widely considered as the juvenile period in rats (36). The concentration of LPS was 0.5 mg/kg (2 ml/kg), as this concentration has been shown to induce an inflammatory response and to activate microglia in male C57BL/6J mice (37). Using a repeated immune activation protocol in female C57BL/6 mice (LPS, *E. coli* strain O111:B4, 1 mg/kg, four injections on alternate days), Shankaran et al. showed increased microglia proliferation and sustained microglial activation even after the last LPS injection [see Ref. (38), reviewed in Ref. (39)]. Our LPS administration protocol has been adapted from these studies. SAL-treated animals received the same number of injections as the LPS-treated animals. D-amphetamine sulfate (AMPH; Fagron) was dissolved in SAL and administered i.p. for the AMPH-induced locomotion test (1.5 mg/kg, 1 ml/kg).

**Figure 1:**
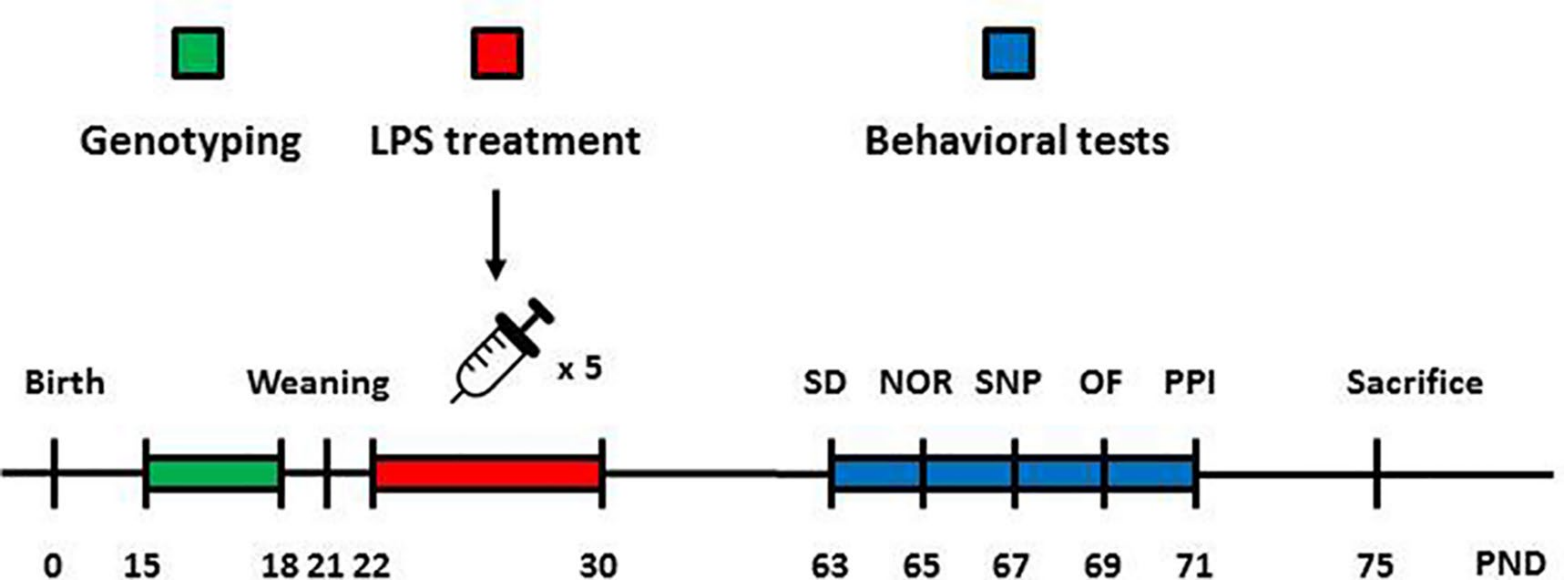
Graphical depiction of the sequence of manipulations and behavioral tests. Animals were genotyped 2 weeks after birth to assess the presence or absence of the knocked-in human DISC1 transgene. Male and female animals without the transgene (wild type) and with the transgene on both alleles (homozygous tgDISC1) were used for behavioral and neurochemical experiments. A day after weaning, animals started receiving LPS or SAL injections (i.p). on alternate days (days 22, 24, 26, 28, and 30). When animals reached adulthood (day 63), behavioral testing began. Animals were tested for SD (day 63), NOR (day 65), SNP (day 67), amphetamine-induced locomotion (day 69), and PPI (day 71). Animals were later sacrificed, and their brains were collected for neurochemical analyses. LPS, lipopolysaccharide; SD, social discrimination; NOR, novel object recognition; SNP, spatial novelty preference; OF, open field; PPI, prepulse inhibition; PND, postnatal day.

Animals were handled and habituated to the test equipment before the experiments began. Separate groups were formed according to sex, genotype, and treatment. Behavioral tests were conducted when animals reached adulthood (9–11 weeks of age) and during the light phase of the light–dark cycle. AMPH-induced locomotion, prepulse inhibition, novel object recognition (NOR), spatial novelty preference (SNP), and social discrimination tests were performed within a 2-week span (**Figure 1**). We used 6–10 animals for each group tested.

### Behavioral Equipment and Procedures

#### Social Discrimination

The social discrimination (SD) test takes advantage of the ability of rats to discriminate between a previously encountered and a novel conspecific. A rectangular gray open field (OF) arena with the dimensions of 100 × 50 × 50 cm was used for testing. During the sample phase, test animals were allowed to explore the arena with two identical wire cup-like restrainers (20 × 20 × 20 cm) on each side of the arena. One restrainer contained a sex- and age-matched animal, whereas the other restrainer was empty. During the choice phase, a novel sex- and age-matched animal was placed in the empty restrainer, and the previously encountered animal was again placed in the same restrainer. The test animals were placed back into the arena for another 5 min. The trials were separated by a 1-h intertrial interval. The scoring of the social behavior was performed afterwards using the videotapes. Interaction was defined as the contact between the test rat and the wire restrainer, as the metal wires were small enough to prevent direct physical contact between animals. Social preference was calculated by the duration of interaction with the animal-containing restrainer compared to the empty restrainer during the sample phase. Social memory was calculated by the duration of interaction with the novel animal-containing restrainer compared to the familiar animal-containing restrainer during the choice phase. Novel and familiar animals as well as their positions were counterbalanced between sessions (40, 41).

#### Novel Object Recognition

A rectangular gray OF arena with the dimensions of 100 × 50 × 50 cm was used for testing. During the sample phase, animals were allowed to explore the arena with two identical objects for 5 min. During the choice phase, one copy of the object was replaced by a novel object, and animals were placed back into the arena for another 5 min. The trials were separated by a 1-h intertrial interval. Scoring of the object exploration was performed afterwards using the videotapes. Exploration was defined as sniffing the object or touching it with the nose. A plastic container filled with marbles and a plastic restrainer were used as objects. Objects were similar in size, but were different in shape, color, and pattern. Between sessions, the objects were wiped with 70% ethanol. The objects were sufficiently heavy and were not displaced by rats. The objects and their positions were counterbalanced between sessions (40, 42).

#### Spatial Novelty Preference

A Y-shaped maze with three arms (60 × 15 × 30 cm) was used for testing. The three arms of the apparatus were separated by 120° angles. Sample and choice phases were separated by an intertrial period of 1 h. During the sample phase, animals were positioned to the start arm and were allowed to explore for 5 min, but the entrance to the novel arm was blocked. During the choice phase, animals were placed back to the start arm and were allowed to explore all three arms (start, novel, familiar) for 2 min. Novel arm duration and visit were compared with familiar arm duration and visit to assess spatial memory performance. Scoring of the arm preference was performed using the videotapes. The blocked arm was counterbalanced between sessions (43).

#### AMPH-Induced Locomotion

A cubic gray OF arena with the dimensions of 50 × 50 × 50 cm was used for testing. The central zone of the arena was defined as a central square with the dimensions of 25 × 25 cm. A low-intensity light (25 lx) was evenly distributed across the OF arena. Firstly, baseline activities of the animals in the arena were assessed for an initial 20 min. Then, animals received an AMPH injection (1.5 mg/kg, i.p)., and their activities were recorded for 60 min. Locomotor and central activities of the animals were analyzed using Biobserve Viewer III software (Biobserve GMBH, Germany; 44, 45).

#### Prepulse Inhibition of the Acoustic Startle Response

Soundproof boxes were used for testing. Acoustic stimuli were presented by two loudspeakers mounted within these boxes. Animals were restrained by metal cages (27 × 9 × 10 cm), which were placed within the boxes. Using piezoelectric accelerometers, acoustic startle response (ASR)-induced physical force was transformed into an electrical signal, which was measured by the TSE startle response system (TSE Systems, Bad Homburg, Germany). The prepulse inhibition (PPI) procedure was adapted from Peleg-Raibstein et al. (46) and established in our previous studies (44). In brief, three prepulse intensities (74, 80, and 86 dB) and three pulse intensities (100, 110, and 120 dB) were used to assess the PPI. Nine prepulse + pulse trials, three pulse-alone trials, three prepulse-alone trials, and one no-stimulus trial were applied in a pseudo-randomized fashion and were repeated 10 times (44, 46, 47). %PPI was calculated with the following formula: %PPI = 100 − [100 × (startle amplitude of prepulse + pulse trials/startle amplitude of pulse-alone trials)].

### Postmortem Monoamine Measurements

After the behavioral tests, animals were sacrificed. Brains were quickly harvested and frozen in a −80ºC freezer. We prepared coronal sections, which were used to dissect the prefrontal cortex and dorsal and ventral striatum. Dissections were performed according to the rat brain atlas (48). The monoamine content in these regions was assessed using high-performance liquid chromatography as previously described (44). In brief, dissected tissues were purified, dissolved in 0.5 M HClO_4_ (Carl Roth, GmbH), and analyzed with electrochemical detection to measure the concentrations of dopamine, serotonin, and noradrenaline. The samples were segregated on a reversed-phase column (ET 125/2, Nucleosil 120-5, C-18; Macherey and Nagel, Dueren, Germany). The working potential of the electrochemical detector (Intro, Antec, Netherlands) was set at 500 mV vs. an ISAAC reference electrode (Antec, Leyden, Netherlands) (44, 49, 50).

### Data Analysis

The data were shown as mean ± SEM. All statistical analyses were carried out with IBM SPSS 21 software (SPSS Inc., Chicago, IL). Statistical significance was set *p* < 0.05 for all tests. Male and female animals were separately analyzed for all tests. Behavioral and neurochemical data were analyzed using ANOVA with genotype and treatment as between-subject factors. Locomotor and central activities in the OF, NOR, SNP, SD, and PPI analyses were analyzed using ANOVA with repeated measures, with time, prepulse intensity and pulse intensity, and choice being within-subject factors. Significant main effects or interactions were followed by preplanned Bonferroni-corrected Fisher’s least significant difference (LSD) comparisons whenever appropriate. 

## Results

### tgDISC1 and Juvenile Immune Activation Interact to Induce a Hyperlocomotor Response to AMPH in Females

**Figure 2:**
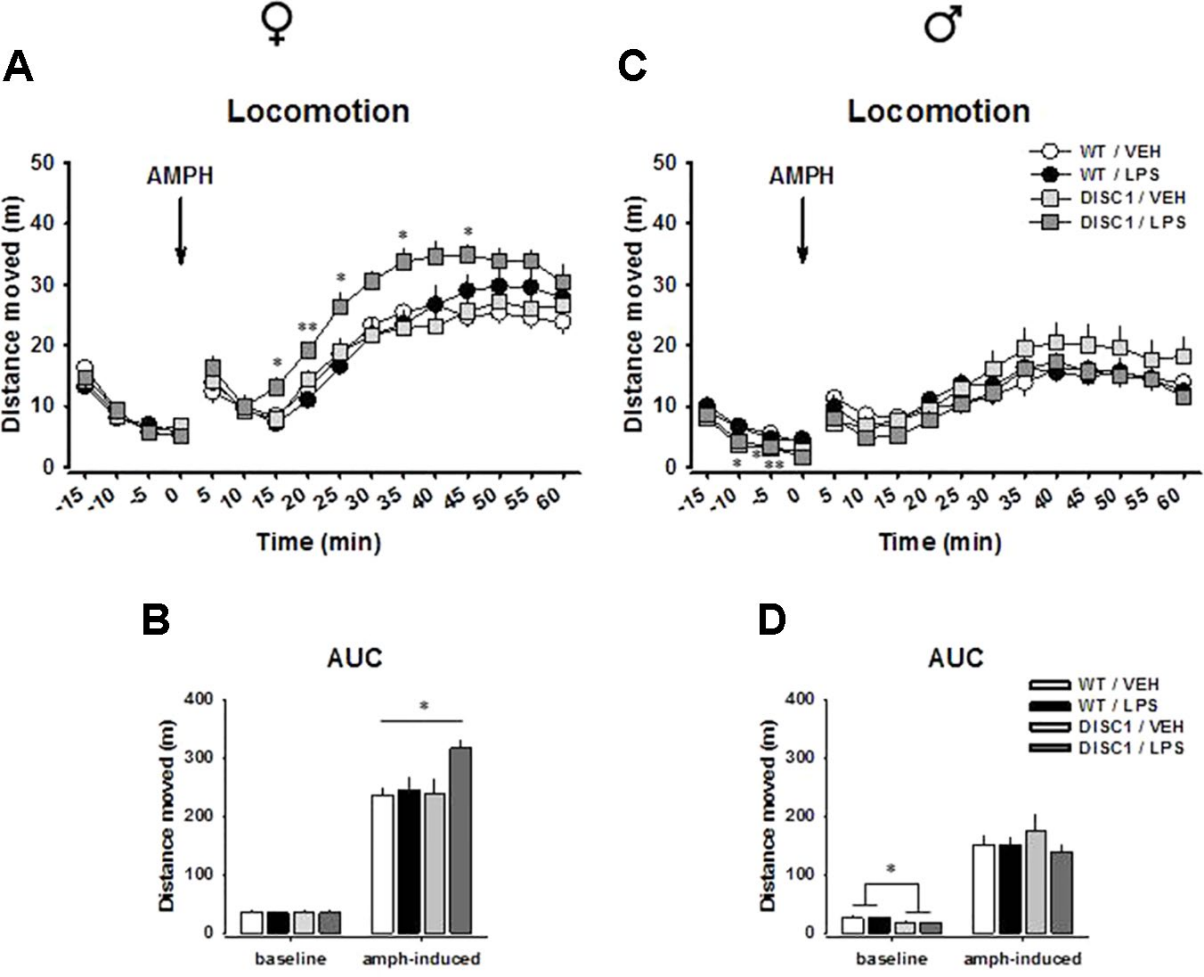
The effects of tgDISC1 with or without juvenile LPS administration on baseline and amphetamine-induced locomotor activity in adult female (left) and male rats (right). Baseline and amphetamine-induced locomotion are shown as activity at single time points for female **(A)** and male rats **(C)**. Baseline and amphetamine-induced locomotion are shown as overall activity for female **(B)** and male rats **(D)**. Arrow represents the amphetamine injection time, which is set to min 0. n = 6–10 per group. Amphetamine challenge dose = 1.5 mg/kg. Values are shown as mean ± SEM. *p < 0.05, **p < 0.01 compared to WT/VEH. WT, wild type; VEH, vehicle; LPS, lipopolysaccharide; AMPH, amphetamine; AUC, area under the curve.

During the 20-min baseline, animals habituated to the test environment. Their locomotor activities declined over time [female: *F*(3,81) = 168.948, *p* < 0.001; male: *F*(3,78) = 53.455, *p* < 0.001]. A significant effect was absent for genotype, LPS treatment, or their interaction for females, suggesting that neither tgDISC1 nor LPS influenced baseline behavior (**Figure 2A and B**). A significant genotype effect, however, was observed in males [*F*(1,26) = 9.023, *p* = 0.006], suggesting reduced baseline locomotor activity induced by tgDISC1 (**Figure 2C and D**). Such a genotype effect, or a strong tendency, was also observed at single time points [min −10: wild type (WT)/vehicle (VEH) vs. DISC1/VEH, *p* = 0.04; min −5: WT/VEH vs. DISC1/VEH, *p* = 0.006; and WT/VEH vs. DISC1/LPS, *p* = 0.012; min 0: WT/VEH vs. DISC1/LPS, *p* = 0.064]. LPS treatment, however, did not influence the baseline locomotor behavior in males.

An AMPH challenge induced an increase in locomotion in all treatment groups for both males and females. For females, we found strong effects of time [*F*(11,297) = 81.142, *p* < 0.001] and treatment [*F*(1,27) = 5.035, *p* = 0.033] and strong tendencies for genotype [*F*(1,27) = 3.338, *p* = 0.079] and time × genotype × treatment interaction [*F*(11,297) = 1.741, *p* = 0.064], suggesting that both tgDISC1 and LPS treatment influenced the locomotor response to an AMPH challenge. Preplanned comparisons suggested that DISC1/LPS animals had an elevated response to AMPH compared to WT/VEH animals, as indicated at single time points (min 15: *p* = 0.036, min 20: *p* = 0.009, min 25: *p* = 0.035; min 35: *p* = 0.027, min 45: *p* = 0.019; **Figure 2A**) and in the area under the curve (AUC) analysis (*p* = 0.023; **Figure 2B**). Such an elevation was absent when tgDISC1 and LPS treatment were not combined (*p* > 0.05), implying that only the combination of genotype and treatment led to an augmented locomotor response to AMPH in female rats. For male rats, we found effects of time [*F*(11,286) = 30.514, *p* < 0.001] and time × genotype interaction [*F*(11,286) = 2.126, *p* = 0.019]; however, there was no effect of genotype, treatment, or their interaction (*p* > 0.05), suggesting that neither tgDISC1 nor LPS treatment influenced AMPH-induced locomotor behavior in male rats (**Figure 2C and D**). We repeated the testing of psychostimulant-induced hyperlocomotion in male tgDISC1 rats in an independent cohort with a 10 mg/kg i.p. cocaine or saline challenge. Also, in this test, cocaine induced a profound hyperlocomotion, which was not affected by the DISC1 genotype in males (**Supplementary Figure S1**). Altogether, these findings advocate sex-specific effects of tgDISC1 and JIA; only the tgDISC1 × JIA combination elevates AMPH-induced locomotor activity in females and tgDISC1 alone reduces the baseline locomotor activity in males.

### Juvenile Immune Activation Disrupts Spatial Memory in Males

**Figure 3:**
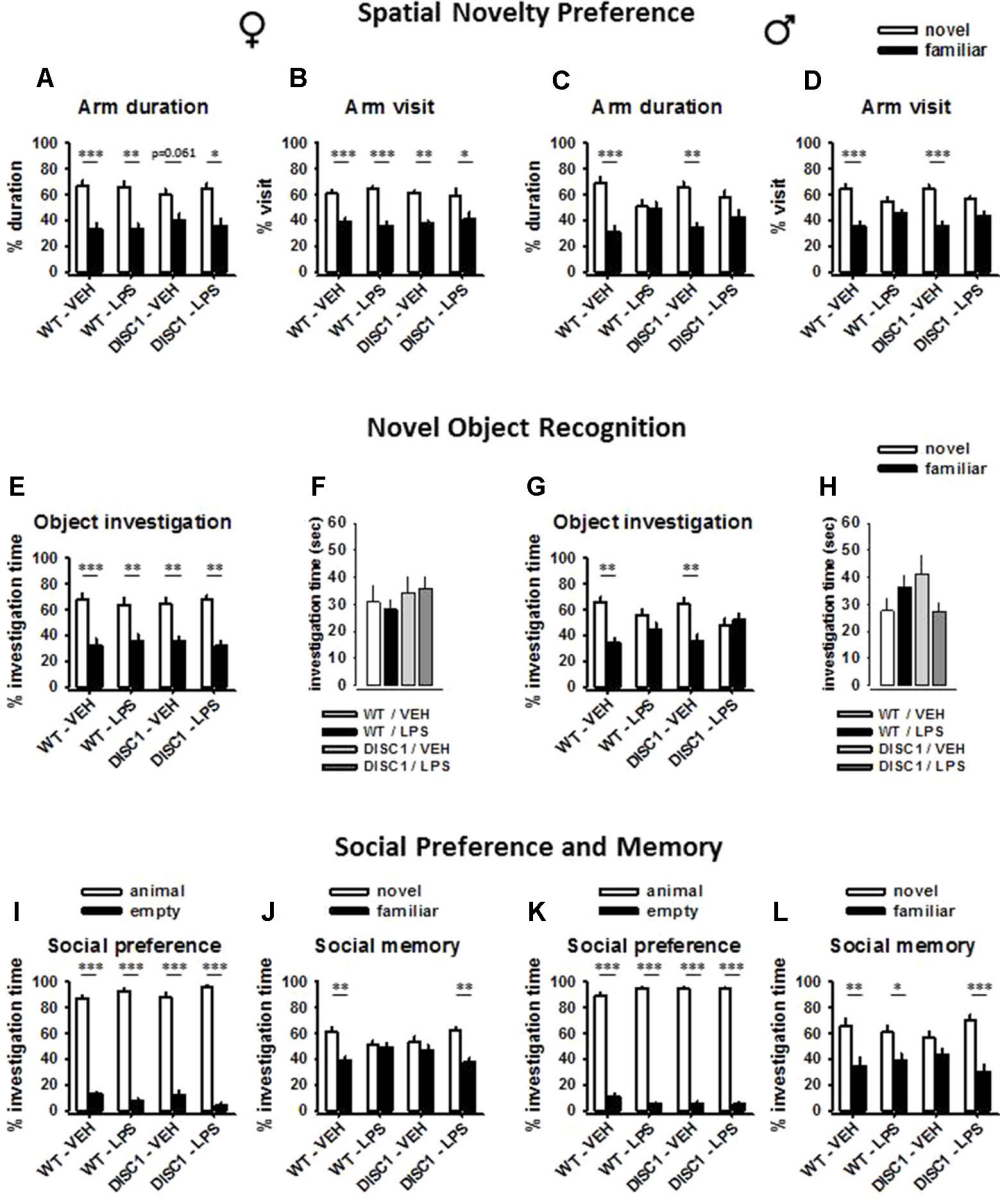
The effects of tgDISC1 with or without juvenile LPS administration on three different aspects of memory performance in adult female (left) and male rats (right). Spatial memory performance, which was tested using a Y-maze, is shown using the parameters of arm duration and arm visit for female **(A** and **B)** and male rats **(C** and **D)**. Recognition memory performance and object exploration time are shown for female **(E** and **F)** and male rats **(G** and **H)**. Social memory performance and social preference are shown for female **(I** and **J)** and male rats **(K** and **L)**. n = 6–10 per group. Values are shown as mean ± SEM. *p < 0.05, **p < 0.01, ***p < 0.001 compared to WT/VEH. WT, wild type; VEH, vehicle; LPS, lipopolysaccharide.

A significant effect of arm choice was observed for female rats [arm duration: *F*(1,27) = 34.978, *p* < 0.001; arm visit: *F*(1,27) = 48.929, *p* < 0.001]. However, this choice was not influenced by either genotype or LPS treatment (*p* > 0.05). Pairwise within-subject comparisons indicate that animals in all groups showed preference towards the novel arm, as they spent more time at the novel arm (WT/VEH: *p* < 0.001; WT/LPS: *p* = 0.002; DISC1/VEH, *p* = 0.061; DISC1/LPS, *p* = 0.013; **Figure 3A**) and they visited it more frequently than the familiar arm (WT/VEH: *p* = 0.001; WT/LPS: *p* < 0.001; DISC1/VEH, *p* = 0.002; DISC1/LPS, *p* = 0.017; **Figure 3B**).

A significant effect of arm choice was observed for male rats [arm duration: *F*(1,26) = 18.818, *p* < 0.001; arm visit: *F*(1,26) = 35.116, *p* < 0.001]. Furthermore, this choice interacted with the LPS treatment [arm duration: *F*(1,26) = 7.161, *p* = 0.013; arm visit: *F*(1,26) = 6.598, *p* = 0.016], but not with the genotype (*p* > 0.05). Pairwise within-subject comparisons indicate that WT untreated animals and tgDISC1 untreated animals showed preference towards the novel arm, as they spent more time at the novel arm (WT/VEH: *p* = 0.001; DISC1/VEH, *p* = 0.005; **Figure 3C**) and they visited it more frequently than the familiar arm (WT/VEH: *p* < 0.001; DISC1/VEH, *p* < 0.001; **Figure 3D**). On the other hand, LPS-treated animals did not show preference towards the novel arm (*p* > 0.05). Altogether, these findings advocate sex-specific effects of JIA, disrupting spatial memory in male but not in female rats. tgDISC1 did not influence the spatial memory performance of either sex.

### Juvenile Immune Activation Disrupts Recognition Memory in Males

A significant effect of object choice was observed for female rats [*F*(1,27) = 43.404, *p* < 0.001]; however, this choice was influenced by neither genotype nor LPS treatment (*p* > 0.05). Pairwise within-subject comparisons indicate that animals in all groups showed preference towards the novel object, as they spent more time investigating it (WT/VEH: *p* < 0.001; WT/LPS: *p* = 0.007; DISC1/VEH, *p* = 0.007; DISC1/LPS, *p* = 0.003; **Figure 3E**). Furthermore, object investigation time during the choice phase was not influenced by either genotype or LPS treatment (*p* > 0.05; **Figure 3F**).

A significant effect of object choice was observed for male rats [*F*(1,26) = 11.586, *p* = 0.002]. Furthermore, this choice interacted with the LPS treatment [*F*(1,26) = 7.261, *p* = 0.012], but not with the genotype (*p* > 0.05). Pairwise within-subject comparisons indicate that WT untreated animals and tgDISC1 untreated animals showed preference towards the novel object, as they spent more time investigating it (WT/VEH: *p* = 0.002; DISC1/VEH, *p* = 0.01; **Figure 3G**). On the other hand, LPS-treated animals (both WT and tgDISC1) did not show preference towards the novel object (*p* > 0.05). Furthermore, object investigation time during the choice phase was not influenced by either genotype or LPS treatment, ruling out a possible effect of motivational deficit for disrupted memory (*p* > 0.05; **Figure 3H**). Altogether, these findings advocate sex-specific effects of JIA, disrupting recognition memory in male but not in female rats. tgDISC1 did not influence the recognition memory performance of either sex.

### tgDISC1 and Juvenile Immune Activation Interact to Restore Social Memory in Females and Males

The comparison of the restrainer with a conspecific and an empty restrainer revealed a significant effect of investigation time for female rats [*F*(1,27) = 988.845, *p* < 0.001]. This preference interacted with the LPS treatment [*F*(1,27) = 6.62, *p* = 0.016], but not with the genotype (*p* > 0.05). Still, pairwise within-subject comparisons revealed that animals in all treatment groups spent more time investigating the restrainer with an animal compared to the empty restrainer (*p* < 0.001; **Figure 3I**). The comparison of the familiar animal with the novel animal revealed a significant effect of investigation time for female rats [*F*(1,27) = 14.212, *p* = 0.001]. This effect was influenced by a genotype × LPS treatment interaction [*F*(1,27) = 6.821, *p* = 0.015]. Pairwise within-subject comparisons indicate that WT/VEH animals discriminated the familiar animal, as they spent less time investigating it compared to the unfamiliar animal (*p* = 0.002; **Figure 3J**). Both tgDISC1 and LPS treatment disrupted the social memory when not combined (*p* > 0.05). Surprisingly, the combination of tgDISC1 and LPS treatment restored the social memory (*p* = 0.007).

The comparison of the restrainer with a conspecific and an empty restrainer revealed a significant effect of investigation time for male rats [*F*(1,26) = 2101.641, *p* < 0.001]. However, this preference was not influenced by either genotype or LPS treatment (*p* > 0.05). Animals in all treatment groups spent more time investigating the restrainer with an animal compared to the empty restrainer (*p* < 0.001; **Figure 3K**). The comparison of the familiar animal with the novel unfamiliar animal revealed a significant effect of investigation time for male rats [*F*(1,26) = 23.029, *p* < 0.001]. However, this effect was not influenced by either genotype or LPS treatment (*p* > 0.05). Pairwise within-subject comparisons indicate that WT/VEH animals discriminated the familiar animal, as they spent less time investigating it compared to the unfamiliar animal (*p* = 0.007; **Figure 3L**). LPS treatment did not influence the social memory performance, as LPS-treated WT animals also spent more time investigating the stranger animal (*p* = 0.047). However, untreated tgDISC1 animals investigated both novel and familiar animals with a similar duration, suggesting disrupted social memory (*p* > 0.05). The combination of tgDISC1 and LPS treatment restored the social memory (*p* = 0.001). Altogether, these findings advocate sex-nonspecific effects of tgDISC1, disrupting social memory without influencing the social preference in both male and female rats. Furthermore, the tgDISC1 × JIA combination restores social memory in both female and male animals.

### tgDISC1 and Juvenile Immune Activation Interact to Induce an Anxiolytic Response to AMPH in Males

**Figure 4:**
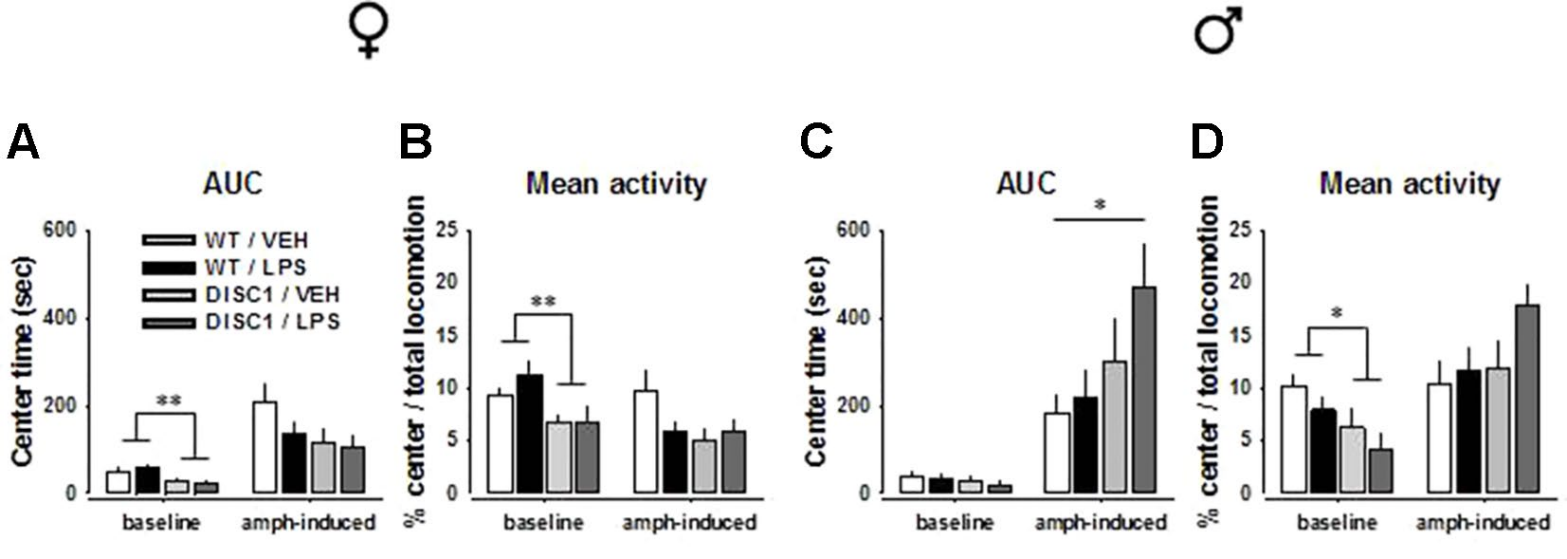
The effects of tgDISC1 with or without juvenile LPS administration on baseline and amphetamine-induced center behavior in the OF as a measure of anxiety in adult female (left) and male rats (right). Baseline and amphetamine-induced center time and center locomotion ratio are shown for female **(A** and **B)** and male rats **(C** and **D)**. n = 6–10 per group. Amphetamine challenge dose = 1.5 mg/kg. Values are shown as mean ± SEM. *p < 0.05, **p < 0.01 compared to WT/VEH. WT, wild type; VEH, vehicle; LPS, lipopolysaccharide; AMPH, amphetamine; AUC, area under the curve.

During the 20-min baseline, there was a significant effect of the genotype [center time: *F*(1,27) = 8.654, *p* = 0.007; center locomotion ratio: *F*(1,27) = 11.078, *p* = 0.003] for female animals, suggesting baseline anxiogenic behavior in female tgDISC1 animals (**Figure 4A and B**). A similar genotype effect was also observed for male animals, but only for the center locomotor ratio [*F*(1,26) = 5.655, *p* = 0.025; **Figure 4C and D**]. However, for both sexes, the lack of a treatment effect or an interaction effect suggests that LPS treatment did not influence the baseline center behavior.

An AMPH challenge induced contrasting effects for female and male animals. For female animals, there was no effect of genotype, LPS treatment, or their interaction (*p* > 0.05). Preplanned Bonferroni-corrected comparisons did not indicate any difference either (**Figure 4A and B**; **Supplementary Figure S2A** and **B**; *p* > 0.05). For male animals, however, a significant effect and a tendency of genotype was observed [center time: *F*(1,26) = 5.508, *p* = 0.027; center locomotion ratio: *F*(1,26) = 3.069, *p* = 0.092]. Preplanned comparisons suggested that unlike the WT/VEH animals, DISC1/LPS animals had an anxiolytic response to AMPH, indicated at the overall level (center time: *p* = 0.049; **Figure 4C and D**) as well as at single time points (**Supplementary Figure S2C** and **D**). Such a response was absent when tgDISC1 and LPS treatment were not combined (*p* > 0.05), implying that only the combination of genotype and treatment led to an anxiolytic response to AMPH in male rats. Altogether, these findings advocate sex-specific effects of tgDISC1 and JIA; only the tgDISC1 × JIA combination led to AMPH-induced anxiolytic behavior in males. On the baseline level, however, tgDISC1 seems to induce an anxiogenic response in both female and male animals.

### Prepulse Inhibition of the Acoustic Startle Response Is Potentiated by tgDISC1 in Females

**Figure 5:**
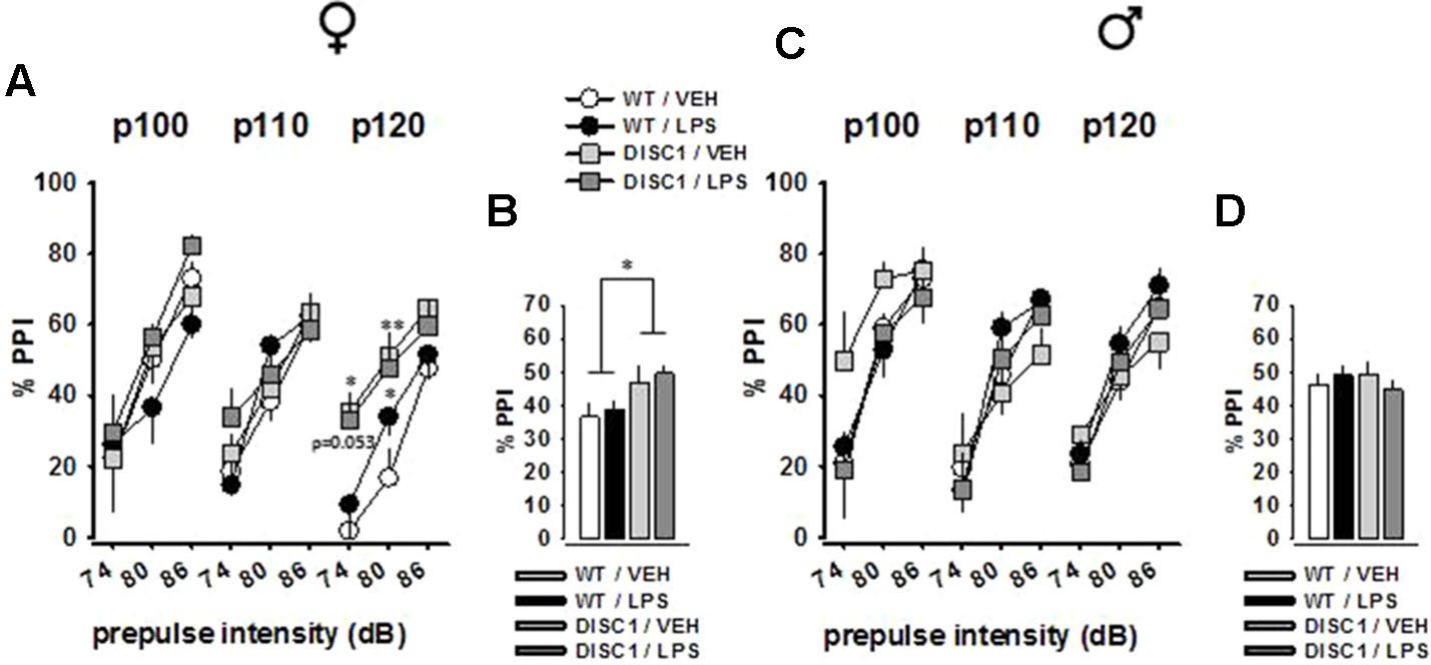
The effects of tgDISC1 with or without juvenile LPS administration on sensorimotor gating in adult female (left) and male rats (right). Prepulse inhibition of the acoustic startle stimulus is shown for each prepulse–pulse pair for female **(A)** and male rats **(C)**. Prepulse inhibition of the acoustic startle stimulus is shown as the overall inhibition for female **(B)** and male rats **(D)**. Background noise = 68 dB. n = 6–10 per group. Values are shown as mean ± SEM. *p < 0.05, **p < 0.01 compared to WT/VEH. WT, wild type; VEH, vehicle; LPS, lipopolysaccharide; PPI, prepulse inhibition; P100, pulse intensity = 100 dB; P110, pulse intensity = 110 dB; P120, pulse intensity = 120 dB.

**Figure 5A and C** shows the mean PPI of animals with all possible prepulse × pulse pairings for female and male animals, respectively. The amount of inhibition increased with increased prepulse stimulus intensity for both sexes, which was evident by a main effect of prepulse level [female: *F*(2,54) = 193.222, *p* < 0.001; male: *F*(2,52) = 152.935, *p* < 0.001]. Furthermore, the effects of pulse level [female: *F*(2,54) = 3.25, *p* = 0.046; male: *F*(2,52) = 3.408, *p* = 0.041], pulse level × genotype interaction [female: *F*(2,54) = 2.668, *p* = 0.079; male: *F*(2,52) = 2.904, *p* = 0.064], and prepulse level × treatment interaction [male: *F*(2,52) = 3.463, *p* = 0.039] were observed. However, a significant genotype effect was only observed in female animals [*F*(2,54) = 6.61, *p* = 0.016], which was a potentiation effect rather than an impairment (**Figure 5B**). This effect was further supported by the analyses of single prepulse–pulse pairings (74–120 dB pairing: WT/VEH vs. DISC1/VEH, *p* = 0.026; WT/VEH vs. DISC1/LPS, *p* = 0.053; 80–120 dB pairing: WT/VEH vs. DISC1/VEH, *p* = 0.006; WT/VEH vs. DISC1/LPS, *p* = 0.019). For male animals, there was no effect of either genotype or LPS treatment (**Figure 5D**). Altogether, these findings suggest a potentiation effect of tgDISC1 on the sensorimotor gating system only in female animals, whereas JIA does not influence this inhibitory response.

### Effects of Juvenile Immune Activation and tgDISC1 on Brain Monoamine Levels in Females

**Figure 6:**
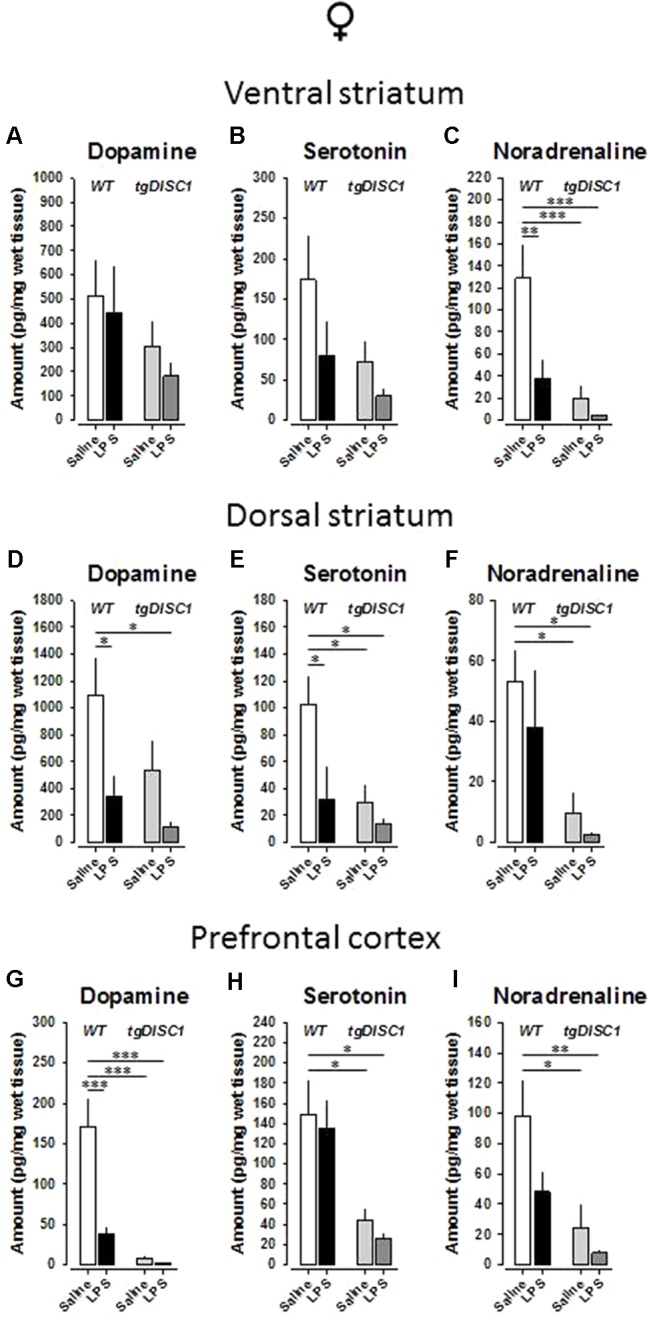
The effects of tgDISC1 with or without juvenile LPS administration on postmortem monoamine levels in adult female rats. Postmortem tissue levels of dopamine **(A**, **D**, and **G)**, serotonin **(B**, **E**, and **H)**, and noradrenaline **(C**, **F**, and **I)** are shown for ventral striatum, dorsal striatum, and prefrontal cortex, respectively. n = 6–10 per group. Values are shown as mean ± SEM. *p < 0.05, **p < 0.01, ***p < 0.001 compared to WT/VEH. WT, wild type; VEH, vehicle; LPS, lipopolysaccharide.

As altered homeostasis of monoamines is a widespread neurochemical abnormality observed in schizophrenia patients (34), we investigated the possible effects of tgDISC1, JIA, and tgDISC1 × JIA on dopamine, serotonin, and noradrenaline levels of the postmortem tissue samples for critical brain regions. In the ventral striatum of female animals, neither dopamine (**Figure 6A**) nor serotonin (**Figure 6B**) levels were influenced (*p* > 0.05). However, noradrenaline level was strongly affected by genotype [*F*(1,26) = 13.967, *p* < 0.001] and treatment [*F*(1,26) = 7.695, *p* = 0.01], and a tendency for their interaction was evident [*F*(1,26) = 4.045, *p* = 0.055; **Figure 6C**]. Preplanned comparisons revealed reduced noradrenaline concentration by LPS (WT/VEH vs. WT/LPS: *p* = 0.005), tgDISC1 (WT/VEH vs. DISC1/VEH: *p* = 0.001), and their interaction (WT/VEH vs. DISC1/LPS: *p* < 0.001).

In the dorsal striatum of female animals, there were significant effects of genotype on the serotonin [*F*(1,28) = 5.465, *p* = 0.027] and noradrenaline [*F*(1,27) = 10.378, *p* = 0.003] levels. Furthermore, significant effects of treatment were evident for dopamine [*F*(1,29) = 6.71, *p* = 0.015] and serotonin [*F*(1,28) = 4.822, *p* = 0.037]. However, no interaction effect was evident for any of the monoamines examined (*p* > 0.05). Preplanned Bonferroni-corrected comparisons revealed reduced dopamine concentration by LPS (WT/VEH vs. WT/LPS: *p* = 0.046; WT/VEH vs. DISC1/LPS: *p* = 0.017; **Figure 6D**), reduced noradrenaline concentration by tgDISC1 (WT/VEH vs. DISC1/VEH: *p* = 0.039; WT/VEH vs. DISC1/LPS: *p* = 0.025; **Figure 6F**), and reduced serotonin concentration by both LPS and tgDISC1 (WT/VEH vs. WT/LPS: *p* = 0.033; WT/VEH vs. DISC1/VEH: *p* = 0.026; WT/VEH vs. DISC1/LPS: *p* = 0.011; **Figure 6E**).

In the prefrontal cortex of female animals, there were significant effects of genotype on dopamine [*F*(1,25) = 30.115, *p* < 0.001], serotonin [*F*(1,28) = 14.254, *p* < 0.001], and noradrenaline [*F*(1,27) = 11.3781, *p* = 0.002] levels. Furthermore, a significant effect of treatment for dopamine [*F*(1,25) = 14.141, *p* < 0.001] and a trend for noradrenaline [*F*(1,28) = 3.874, *p* = 0.059] were evident. A significant interaction effect was also evident for dopamine [*F*(1,25) = 12.198, *p* = 0.002]. Preplanned comparisons revealed reduced serotonin and noradrenaline concentrations by tgDISC1 (serotonin: WT/VEH vs. DISC1/VEH: *p* = 0.033; WT/VEH vs. DISC1/LPS: *p* = 0.015; **Figure 6H**; noradrenaline: WT/VEH vs. DISC1/VEH: *p* = 0.011; WT/VEH vs. DISC1/LPS: *p* = 0.004; **Figure 6I**) and reduced dopamine concentration by both LPS and tgDISC1 (WT/VEH vs. WT/LPS: *p* < 0.001; WT/VEH vs. DISC1/VEH: *p* < 0.001; WT/VEH vs. DISC1/LPS: *p* < 0.001; **Figure 6G**). Altogether, these findings suggest that JIA and tgDISC1 induce region- and monoamine-specific disruptions on the postmortem monoamine concentrations for female animals. At the gross level, monoamine content in the analyzed regions is reduced by both tgDISC1 and JIA.

### Effects of Juvenile Immune Activation and tgDISC1 on Brain Monoamine Levels in Males

**Figure 7:**
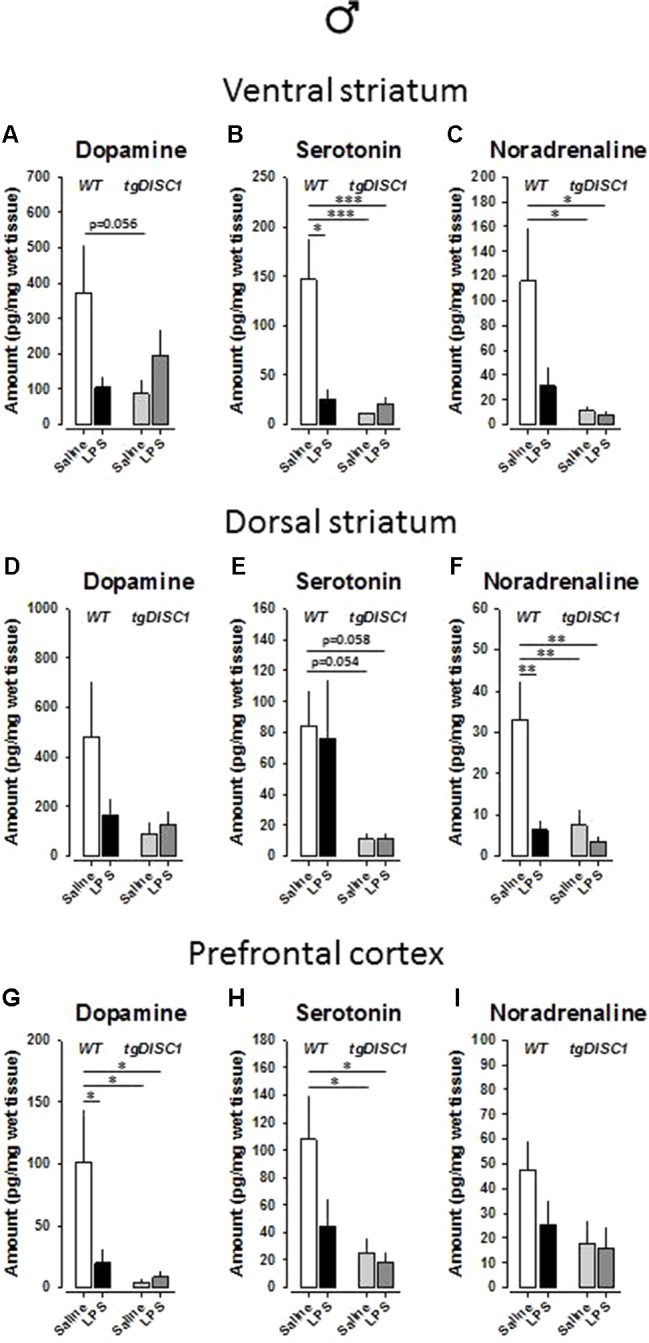
The effects of tgDISC1 with or without juvenile LPS administration on postmortem monoamine levels in adult male rats. Postmortem tissue levels of dopamine **(A**, **D**, and **G)**, serotonin **(B**, **E**, and **H)**, and noradrenaline **(C**, **F**, and **I)** are shown for ventral striatum, dorsal striatum, and prefrontal cortex, respectively. n = 7–10 per group. Values are shown as mean ± SEM. *p < 0.05, **p < 0.01, ***p < 0.001 compared to WT/VEH. WT, wild type; VEH, vehicle; LPS, lipopolysaccharide.

In the ventral striatum of male animals, there were significant effects of genotype on serotonin [*F*(1,26) = 9.548, *p* = 0.005] and noradrenaline [*F*(1,26) = 6.804, *p* = 0.015] levels. Furthermore, a significant effect of treatment was evident for serotonin [*F*(1,26) = 5.992, *p* = 0.021]. Significant interaction effects were also evident for dopamine [*F*(1,26) = 5.11, *p* = 0.032] and serotonin [*F*(1,26) = 8.338, *p* = 0.008]. Preplanned Bonferroni-corrected comparisons revealed reduced dopamine and noradrenaline concentrations by tgDISC1 (dopamine: WT/VEH vs. DISC1/VEH: *p* = 0.056; **Figure 7A**; noradrenaline: WT/VEH vs. DISC1/VEH: *p* = 0.023; WT/VEH vs. DISC1/LPS: *p* = 0.016; **Figure 7C**) and reduced serotonin concentration by both LPS and tgDISC1 (WT/VEH vs. WT/LPS: *p* = 0.014; WT/VEH vs. DISC1/VEH: *p* < 0.001; WT/VEH vs. DISC1/LPS: *p* = 0.001; **Figure 7B**).

In the dorsal striatum of male animals, dopamine level was not influenced (*p* > 0.05; **Figure 7D**). However, there were significant effects of genotype on serotonin [*F*(1,27) = 10.855, *p* = 0.003] and noradrenaline [*F*(1,26) = 6.435, *p* = 0.018] levels. Furthermore, significant effects, or near-significant tendencies, of treatment and treatment × genotype interaction were evident for noradrenaline [treatment: *F*(1,26) = 7.443, *p* = 0.011; treatment × genotype: *F*(1,26) = 4.134, *p* = 0.052]. Preplanned Bonferroni-corrected comparisons revealed strong trends for reduced serotonin concentration by tgDISC1 (WT/VEH vs. DISC1/VEH: *p* = 0.054; WT/VEH vs. DISC1/LPS: *p* = 0.058; **Figure 7E**) and significantly reduced noradrenaline concentration by both LPS and tgDISC1 (WT/VEH vs. WT/LPS: *p* = 0.01; WT/VEH vs. DISC1/VEH: *p* = 0.007; WT/VEH vs. DISC1/LPS: *p* = 0.002; **Figure 7F**).

In the prefrontal cortex of male animals, noradrenaline level was not influenced (*p* > 0.05; **Figure 7I**). However, there were significant effects of genotype on dopamine [*F*(1,28) = 5.91, *p* = 0.022] and serotonin [*F*(1,26) = 6.804, *p* = 0.015] levels. Furthermore, robust tendencies of treatment effect and treatment × genotype interaction were evident for dopamine [treatment: *F*(1,28) = 2.995, *p* = 0.095; treatment**× genotype: *F*(1,28) = 3.726, *p* = 0.064]. Preplanned Bonferroni-corrected comparisons revealed significantly reduced serotonin concentration by tgDISC1 (WT/VEH vs. DISC1/VEH: *p* = 0.023; WT/VEH vs. DISC1/LPS: *p* = 0.016; **Figure 7H**) and significantly reduced dopamine concentration by both LPS and tgDISC1 (WT/VEH vs. WT/LPS: *p* = 0.045; WT/VEH vs. DISC1/VEH: *p* = 0.014; WT/VEH vs. DISC1/LPS: *p* = 0.019; **Figure 7G**). Altogether, these findings suggest that JIA and tgDISC1 induce region- and sex-specific disruptions on brain monoamine concentrations. At the gross level, monoamine content in the analyzed regions is reduced by both tgDISC1 and JIA.

## Discussion

In this experiment, we aimed to test the two-hit model of schizophrenia using genetic and environmental manipulations (51). For this purpose, we investigated the effects of JIA and tgDISC1, in both combined and uncombined situations, on the induction of schizophrenia-related behavioral and neurochemical disruptions in adult female and male rats. We examined possible behavioral aberrations along three major symptom dimensions of schizophrenia including psychosis, social and emotional disruptions, and cognitive impairments. In female animals, we observed significant gene × environment (G × E) interactions in psychotic-like and social behavior, yet the interaction induced rather an improvement of social memory. DISC1 overexpression alone induced anxiogenic behavior and an improvement in sensorimotor gating. Neither tgDISC1 nor JIA influenced spatial memory or recognition memory in female rats under the conditions tested. In male animals, we observed significant G × E interaction in AMPH-induced anxiety behavior and social memory, yet the interaction induced rather improvements in both behaviors. JIA alone disrupted spatial memory and recognition memory. tgDISC1 alone reduced the baseline locomotor behavior. Neither tgDISC1 nor JIA influenced AMPH-induced psychotic behavior, sensorimotor gating, or social preference. We here report rather heterogeneous behavioral phenotypes in terms of sex, treatment, and genotype effects. Such a heterogeneous profile has also been encountered in the postmortem monoamine levels of animals. Our behavioral and neurochemical findings are summarized in **Table 1**.

**Table 1:** The summary of behavioral and neurochemical effects of tgDISC1, JIA, and their combination. ↔, no change; ↑, increase; ↓, decrease; JIA, juvenile immune activation; AMPH, amphetamine; OF, open field; VS, ventral striatum; DS, dorsal striatum; PFC, prefrontal cortex.

	JIA	tgDISC1	JIA x tgDISC1	
**Behavioral Tests**				**Relevance to schizophrenia**
AMPH-induced locomotion	↔	↔	♀↑	psychosis
Novel object recognition	♂↓	↔	no interaction	cognitive deficit
Spatial novelty preference	♂↓	↔	no interaction	cognitive deficit
Social preference / memory	♀↓(memory)	♀♂↓ (memory)	♀♂↑ (memory)	social dysfunction
Center activity in OF	↔	♀♂↓ (baseline)	♂↑ (AMPH-induced)	comorbid anxiety disorder
Prepulse inhibition	↔	♀↑	no interaction	sensory information filtering
**Post mortem neurochemistry**				
Dopamine	♀♂↓ PFC, ♀↓ DS	♀♂↓ PFC	♀↓ PFC	
Serotonin	♀↓ DS, ♂↓ VS	♀♂↓ gross level	no interaction	
Noradrenaline	♀↓ VS, ♂↓ DS	♀♂↓ gross level	no interaction	

Since the identification of a dominant negative mutation in the DISC1 gene of a Scottish family that suffered from various psychiatric disorders (52), it has been heavily investigated and a role of the DISC1 protein in neurodevelopment, synaptic maintenance, and adaptive behavior has been firmly established. DISC1 is an intracellular hub protein that interacts with many other proteins, and these interactions are critical for the proliferation and migration of neurons, as well as dendritic spine regulation and synaptic maintenance during neurodevelopment (3–5). By manipulating the DISC1 gene *via* various approaches in preclinical animal models, including different point mutations and dominant negative models, schizophrenia-related behavioral, anatomical, and neurochemical aberrations have been reported, although these aberrations were mild, subtle, and sometimes variable depending on the manipulation technique of the DISC1 gene (22). Different mutant DISC1 models induced variable social behavior in mice (53–55). DISC1 overexpression did not influence social preference in rats. However, social memory was disrupted in tgDISC1, in both male and female rats, thus supporting a report by Haque et al. for Disc1-L100P +/- male mice (56). Furthermore, some two-hit model studies indicated synergistic effects of tgDISC1 and prenatal immune activation to impair social behavior [(55); dominant negative DISC1 (57); Disc1-Q31L +/-]. In contrast to these studies, we found that synergistic interaction of JIA and tgDISC1 improved social memory in both female and male animals. Behaviors such as social motivation, pair bonding, social recognition, and discrimination of social stimuli are generally orchestrated by the neuromodulator oxytocin. tgDISC1-induced social memory disruptions could be rescued by a compensatory activation of the oxytocin system by JIA, although this speculative notion remains to be investigated.

Schizophrenia patients exhibit cognitive disruptions including deficits in attention, executive functioning, and short-term memory. Furthermore, schizophrenia patients have been shown to have reduced spine density on hippocampal and cortical pyramidal neurons, implicating a potentiation of synaptic pruning during critical neurodevelopmental periods to possibly cause these cognitive deficits in early adulthood (8). tgDISC1 did not influence either spatial memory or recognition memory in either sex, similar to previous reports using male mice [(55); dominant negative DISC1 (57); Disc1-L100P +/-] or rats (25). In that, the previously observed deficits in attention/short-term working memory in male tgDISC1 rats (58) may not have affected these types of learning and memory. However, we observed disruptive effects of JIA in both spatial memory and recognition memory in male animals. Microglial activation during juvenile period by LPS caused irreversible cognitive deficits by overpruning of hippocampal and cortical neurons, which manifested their effects in adult male rats. Given the neuroprotective effects of estrogens against the cognitive impairments of schizophrenia (59, 60), it is not a surprise that our LPS-treated female animals did not exhibit memory deficits, whereas LPS-treated male animals did. Reduced LPS-induced microglial activation and/or increased BDNF synthesis by estrogens (61, 62), especially in the cortical and hippocampal regions, possibly protected our female animals from overpruning-induced cognitive deficits. These findings also fit the reports from human studies, which show female schizophrenic patients to be less severely affected by the cognitive symptoms of schizophrenia than male patients (63).

Attenuation of the PPI is widely regarded as an endophenotype of schizophrenia, showing a high translational appeal (64). Thus, the improvement in PPI by tgDISC1 was rather unexpected. Furthermore, this improvement, which was only detected in females, did not interact with JIA. The improvement in tgDISC1 female animals was more pronounced by the highest pulse intensity (120 dB), hinting that a more stressful condition unveils the improvement in PPI. The literature contains mixed findings about the effects of tgDISC1 models on PPI, as both attenuated PPI (56, 57) and unaltered PPI (55, 65) were reported in male tgDISC1 animals. Female tgDISC1 animals have been less commonly tested, and these studies reported unaltered PPI (53, 66, 67). Despite the fact that our DISC1 manipulation model, PPI paradigm, and the subject species are different from these studies, our PPI findings require further validation.

DISC1 protein is involved not only in proper spine formation in prefrontal glutamatergic neurons, but also in modulating certain signaling pathways in striatal dopaminergic neurons, as well as neurodevelopment of the dopamine system (24, 68). Psychosis has been associated with striatal over-activation of both D2-receptor-mediated canonical and noncanonical pathways, both of which have been regulated by antipsychotic drugs (44, 69). DISC1 protein has been shown to influence the noncanonical D2-receptor-mediated pathway in the striatum, by interacting with glycogen synthase kinase-3, as well as with the dopamine D2 receptor itself (53, 68, 70). Furthermore, using the same tgDISC1 model as ours, Trossbach et al. demonstrated elevated dopamine transporter levels and elevated portions of D2 high receptor, a high-affinity form of D2 receptor, in the dorsal striatum (25), suggesting an altered dopaminergic state in those animals. Some cognitive deficits in these animals could be reversed by intranasal DA application (71). Immune activation has also been shown to induce an altered dopaminergic state in the mesolimbic areas of the brain by increasing tyrosine hydroxylase levels in rodents (72). Therefore, the hypersensitivity to AMPH in our female tgDISC1 × JIA animals could be caused by a synergistic action of tgDISC1 and JIA on dopaminergic tone as suggested by the two-hit hypothesis of schizophrenia. Hypersensitivity to psychostimulants in animal models is widely considered to mimic psychosis in patients. Importantly, our postmortem neurochemistry findings failed to detect a tgDISC1 × JIA interaction in the striatal dopamine levels of female animals. Hypersensitivity to psychostimulants has also been observed in a dominant negative tgDISC1 × adolescent stress model in both male and female mice, where they did not observe an interaction in the striatal dopamine levels either (67). In that, also schizophrenia-related behavioral disturbances may not be explained by a rather simple alteration of postmortem monoamine tissue levels. Additional investigations utilizing *in vivo* microdialysis and/or dopamine D2 receptor expression assays could provide useful information regarding functional changes in dopaminergic neurotransmission.

Despite the fact that monoamine tissue levels cannot be directly linked to schizophrenia-related psychopathology, various monoaminergic alterations have been associated with schizophrenia (34). Our postmortem monoamine measurements indicated reduced dopamine content in the prefrontal cortex by both tgDISC1 and JIA, detected in both sexes. An interaction effect was also observed for females. These findings are consistent with previous reports, as reduced frontal dopamine content has also been observed in a dominant negative DISC1 mouse model (35), as well as in the aforementioned dominant negative tgDISC1 × adolescent stress model (67). Furthermore, *in utero* knockdown of DISC1 has been shown to disturb mesocortical dopaminergic projections in mice (73). Therefore, reduced prefrontal dopaminergic neurotransmission, which is repeatedly reported in various DISC1 models as well as in DISC1 G × E interaction models, could underlie the emotional and cognitive disruptions in schizophrenia, fitting well to the dopamine hypothesis of schizophrenia (74). At a gross level, both serotonin and noradrenaline tissue levels have been disturbed by tgDISC1 and, to a certain degree, by JIA. Serotonin signaling in the striatum has been implicated in locomotion and mood control, whereas both prefrontal serotonin and noradrenaline modulate higher-order cognitive functions as well as emotional impairments associated with schizophrenia (75–77). Therefore, alterations in these monoamine systems possibly contributed to the behavioral aberrations detected in this study, which correspond to all three major symptom dimensions of schizophrenia. However, further experimental clarifications and replications are required to associate these alterations with the behavioral phenotype.

Our findings from the G × E model can also be expanded by future studies. Importantly, in order to attribute the reported behavioral changes to aberrations in synaptic pruning, prefrontal and hippocampal spine morphology should be investigated in the postmortem brain tissue. Previous studies found that various mutant DISC1 models show reduced cortical and hippocampal spine density (22, 54, 73, 78, 79), which we would expect to detect in our study. Still, the absence of the confirmation of possible altered spine morphology by our G × E model could be considered a limitation in our study. An interesting investigation utilizing our model could be the measurement of expression levels of critical proteins from the non-canonical dopamine D2-receptor-mediated pathway, glycogen synthase kinase-3 and protein kinase B (Akt), in striatal regions in order to investigate the underlying factors of psychosis. Moreover, oxytocin and/or oxytocin receptor expression in hippocampus, as well as the interaction of oxytocin system with the microglia during neurodevelopment, could be investigated. This could explain the surprising improvement of social interaction and memory in tgDISC1 × JIA animals.

## Conclusions

Our findings indicate that overexpressed human DISC1 with schizophrenia-related polymorphisms × JIA by the LPS model is a useful tool that can be used in future studies to investigate the two-hit, synaptic pruning hypothesis of schizophrenia. The sex-specific behavioral phenotype that we observed here is in line with the sex-biased symptomology of schizophrenia. Future studies should focus more on the factors that influence brain maturation during extremely vulnerable juvenile and early adolescent periods. Such challenges, as indicated here, can interact with the genetic susceptibility factors to synergistically induce schizophrenia-related disruptions in adulthood.

## Ethics Statement

All experiments were conducted in conformity with the Animal Protection Law of the Federal Republic of Germany as well as the European Communities Council 2010 Directive (2010/63/EU) and were approved by local authorities (Regierung von Mittelfranken).

## Author Contributions

CM, TU, and JH designed the study. CK generated the transgenic rat line. TU, JS, AW, IZ, and MD conducted behavioral experiments. GK performed the neurochemical measurements. TU, AW, and CM did the statistical analysis and wrote the first draft of the manuscript. SvH, JK, JH, and CK revised the manuscript. All authors contributed to and approved the final form of the manuscript.

## Funding

This work was supported by the German Research Foundation (DFG) grant MU 2789/7-1.

## Conflict of Interest Statement

The authors declare that the research was conducted in the absence of any commercial or financial relationships that could be construed as a potential conflict of interest.
