# Supplementary material for: Disrupted-in-Schizophrenia 1 (DISC1) Overexpression and Juvenile Immune Activation Cause Sex-Specific Schizophrenia-Related Psychopathology in Rats
Source: Front Psychiatry. 2019 Apr 9;10:222. doi: 10.3389/fpsyt.2019.00222 (PMC6465888; doi:10.3389/fpsyt.2019.00222)
Supplement: Supplementary file 1 [file DataSheet_1.pdf]

Supplementary material

Material and methods

Animals

Male Sprague Dawley rats which express the full-length non-mutant human DISC1 gene (tgDISC1) and littermate controls (WT) were obtained from the local breeding facility (ZETT, Heinrich-Heine University, Düsseldorf, Germany). Animals, n=12 per group with ages of 9-11 months were grouped 2-3 per cage and housed in Makrolon cages (Type IV; 60 × 38 × 20 cm) with standard temperature and humidity conditions under a reversed 12h light-dark cycle (lights on at 19:00h). They were allowed over two weeks to adapt to the environment and were handled for 5 min/rat/day for 10 consecutive days, with food and water *ad libitum* until the end of the experiments. This study was approved by the Landesamt für Natur, Umwelt und Verbraucherschutz (LANUV) NRW and followed the "Principles of laboratory animal care" (NIH publication No. 86-23, revised 1985) and the German Law on the Protection of Animals.

Apparatus

A black open field made of wood (60 × 60 × 40 cm) was used. Four LED lights were situated above the apparatus, providing illumination of corners and the center (~5 lx). Two geometric symbols were attached to the walls as spatial cues. A camera was hung 2 m above the open field and connected to a computer and a DVD player for analysis and recording.

Drugs

Cocaine hydrochloride (Sigma-Aldrich, Steinheim, Germany) was dissolved in 0.9% saline to a concentration of 10 mg/kg (Müller et al., 2007; Pum et al., 2007) and injected in a volume of 1 ml/kg, intraperitoneally. The drug was prepared fresh before usage.

Behavioral testing

Animals were subjected to a series of behavioral tests including the open field, spontaneous alternation T-maze and the elevated plus-maze and a cocaine-challenge test. To increase the activity level of the animals, they were

food deprived (15 g/rat/day) for one week before the open field test. The animals were placed in a waiting room for about one hour before the beginning of each behavioral test.

Open field habituation

Animals were placed into the open field for 10 min for measuring locomotor activity. Distance moved (cm), velocity (cm/s), frequency and duration (s) of grooming and rearing, and center time in seconds (s) were recorded.

Cocaine-challenge

On day 1 of the cocaine test, the animals were injected with the saline vehicle for estimating the locomotor baseline. The test started immediately after the animals were placed into the open field and lasted for 30 minutes. Twenty-four hours later (day 2), the animals were injected with cocaine and re-exposed to the open field for 30 min. Ten days later, the procedure of cocaine injection and locomotor testing was repeated. Distance moved (cm) was recorded.

Statistics

Mixed two-way ANOVAs with the within factor "distance" and the between factor "genotype" were used to analyze the results in the test of cocaine-challenge. Values represented mean ± SEM. The significant level was set as $p<0.05$.

Results

tgDISC1 does not induce hyperlocomotor response to either saline or cocaine in males

In this work, we attempted to induce the schizophrenic-like behavior via a cocaine-challenge. For the analyses of the main effect or interaction between the within-subjects variable, “distance in 30 min after treatments”, and the between-subjects factor, “genotype”, mixed two-way ANOVAs were used. We obtained a significant main effect of “distance” ($F(2,44) = 28.566$, $P = 0.0001$), but no effect of “genotype” ($F(1,22) = 1.003$, $P = 0.327$) and “distance × group” ($F(2,44) = 0.816$, $P = 0.449$). Afterward, distance moved was divided into two subgroups, the 1st 15 min and the 2nd 15 min. In both subgroups, the significant main effects of “distance” (1st 15 min: ($F(2,44) = 26.869$, $P = 0.0001$; 2nd 15 min: $F(2,44) = 25.769$, $P = 0.0001$), but not “genotype” (1st 15

min: $F(1,22) = 1.264$, $P = 0.273$; 2nd 15 min: $F(1,22) = 0.575$, $P = 0.456$) and “distance × genotype” (1st 15 min: $F(2,44) = 0.470$, $P = 0.628$; 2nd 15 min: $F(2,44) = 1.415$, $P = 0.254$) were found. We attempted to further establish the difference between the WT group and the tgDISC1 genotype group in each treatment via independent-sample *t* tests. Distance moved in total 30 min (Figure S1A) and in every 15 min (Figure S1B) on saline-, first cocaine (cocaine)- and second cocaine (cocaine2)-treatment days were analyzed. No significant difference either in 30-min analysis or every-15-min analysis ($P > 0.05$) was observed. Hence, the cocaine treatment may not increase the locomotion in the tgDISC1 animals.

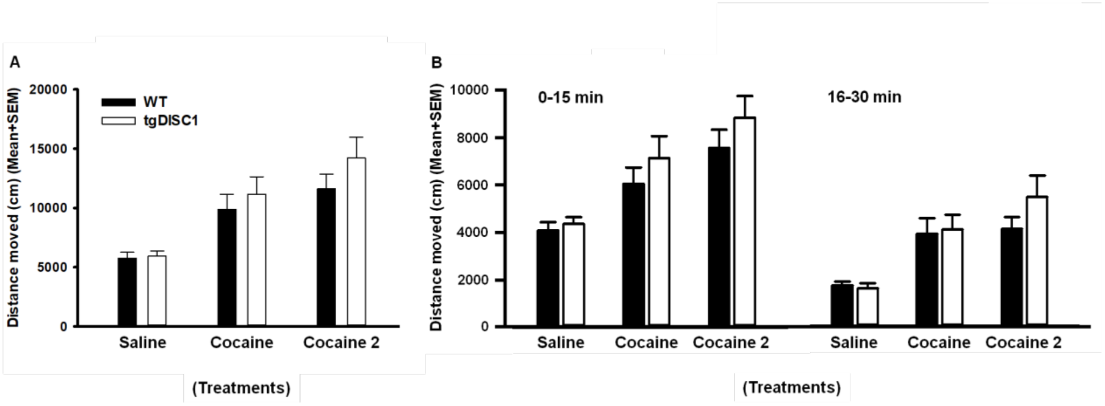

Figure S1: Locomotion in the cocaine-challenge experiment. Distance moved in total duration, 30 min (A), and in every 15 min (B) on saline-, first cocaine-(cocaine) and second cocaine (cocaine 2)-treatment days were computed via independent-sample *t* tests. WT group and tgDISC1 group showed no difference in locomotion after each treatment (n=12/group). Values are represented as mean ± SEM.

tgDISC1 and JIA interact to induce an anxiolytic response to AMPH in males

During the 20 min baseline, center activity was not influenced by time for either sex ($p>0.05$). At single time points, a reduction in center locomotion ratio was observed in DISC1/LPS males (min -5: $p=0.022$; Figure S2D). An AMPH challenge induced contrasting effects for female and male animals. For female animals, there was an effect of time (center time: $F(11,297)=3.519$, $p<0.001$; center locomotion ratio: $F(11,297)=3.739$, $p<0.001$), however, there was no effect of genotype, LPS treatment, or their interaction ($p>0.05$). Single time point comparisons indicated subtle effects of genotype and treatment (center time: min 25: WT/VEH vs DISC1/VEH, $p=0.029$; WT/VEH vs DISC1/LPS, $p=0.06$; min 50: WT/VEH vs WT/LPS, $p=0.059$; WT/VEH vs DISC1/VEH, $p=0.079$; center locomotion ratio: min 5: WT/VEH vs WT/LPS, $p=0.047$; min 30: WT/VEH vs DISC1/VEH, $p=0.06$; WT/VEH vs DISC1/LPS, $p=0.054$; min 50: WT/VEH vs DISC1/VEH, $p=0.055$; Figure S2A, B). For male animals, however, significant effects, or strong tendencies, of time (center time: $F(11,286)=4.09$, $p<0.001$; center locomotion ratio: $F(11,286)=8.518$, $p<0.001$), time x genotype interaction (center time: $F(11,286)=2.095$, $p=0.021$; center locomotion ratio: $F(11,286)=1.649$, $p=0.085$), time x treatment interaction (center time: $F(11,286)=1.965$, $p=0.032$; center locomotion ratio: $F(11,286)=2.794$, $p=0.002$), and genotype (center time: $F(1,26)=5.508$, $p=0.027$; center locomotion ratio: $F(1,26)=3.069$, $p=0.092$) were observed. Single time point comparisons suggested that unlike the WT/VEH animals, DISC1/LPS animals had an anxiolytic response to AMPH (center time: min 30: $p=0.087$, min 35: $p=0.01$, min 40: $p=0.014$; min 45: $p=0.045$; central locomotion ratio: min 35: $p=0.042$, min 40: $p=0.02$; min 45: $p=0.038$; min 60: 0.055; Figure S2C, D). Such a response was absent when tgDISC1 and LPS treatment were not combined ($p>0.05$), implying that only the combination of genotype and treatment led to an anxiolytic response to AMPH in male rats.

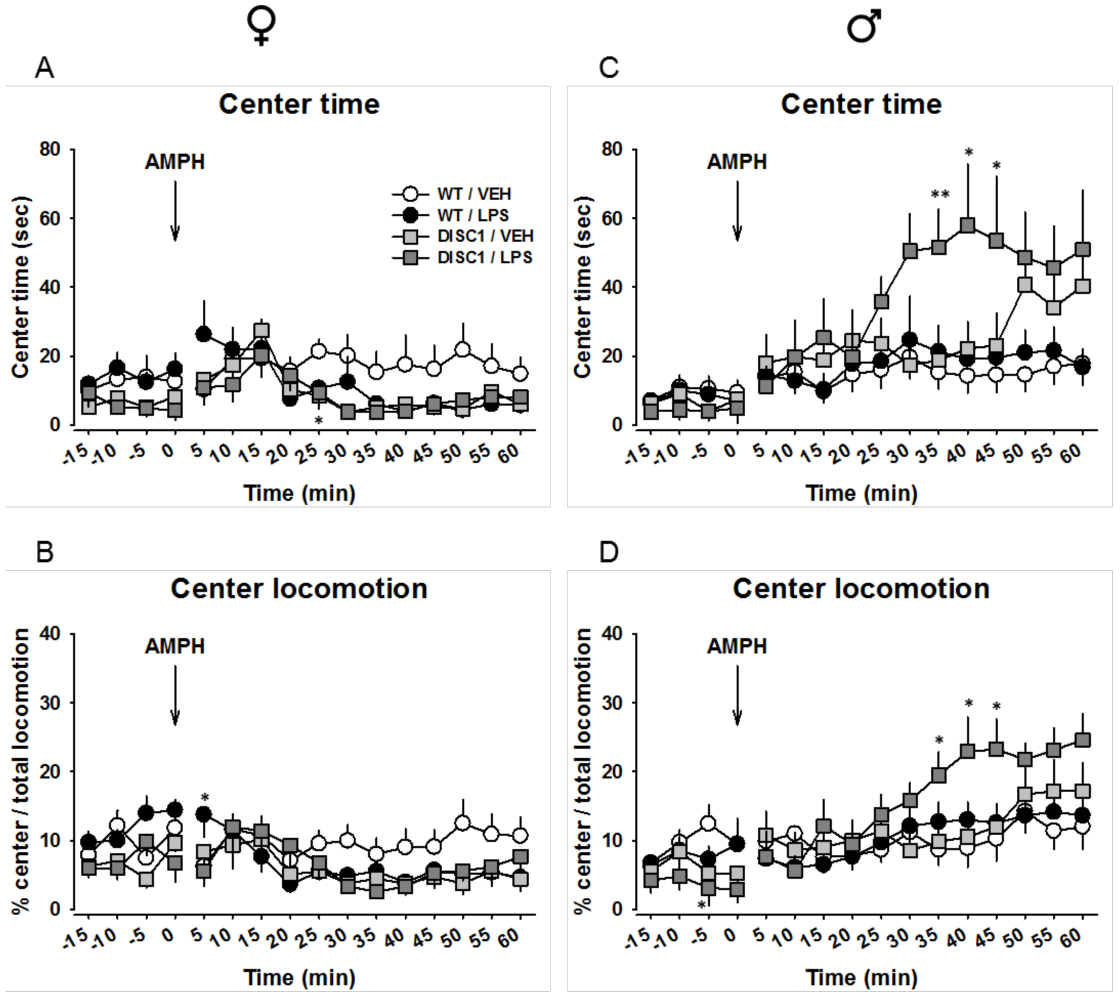

Figure S2: The effects of tgDISC1 with or without juvenile LPS administration on baseline and amphetamine-induced center behavior in adult female (left) and male rats (right), shown at single time points. Arrow represents the amphetamine injection time, which is set to min 0. n = 6-10 per group. Amphetamine challenge dose = 1.5 mg/kg. Values are shown as mean ± SEM. * $p < 0.05$, ** $p < 0.01$ compared to WT/VEH. WT, wild type; VEH, vehicle; LPS, lipopolysaccharide; AMPH, amphetamine.

References

Müller CP, de Souza Silva MA, Huston JP. Double dissociating effects of sensory stimulation and cocaine on serotonin activity in the occipital and temporal cortex. *Neuropharmacology* (2007) 52:854-62.

Pum M, Carey RJ, Huston JP, Müller CP. Dissociating effects of cocaine and d-amphetamine on dopaminergic and serotonergic activity in the entorhinal, perirhinal and prefrontal cortex: an in-vivo microdialysis study. *Psychopharmacology* (2007) 193(3):375-90.
